# Relaxin‐2 improves type I diabetes mellitus‐induced erectile dysfunction in rats by protecting cavernous endothelial and smooth muscle function, and inhibiting penile fibrosis and apoptosis

**DOI:** 10.1111/andr.13822

**Published:** 2024-12-09

**Authors:** Bocheng Tu, Kang Liu, Bo Wen, Peng Hu, Taotao Sun, Beining Li, Manan Sulaiman, Shujun Jiang, Tao Wang, Jihong Liu, Yang Luan

**Affiliations:** ^1^ Department of Urology Tongji Hospital Tongji Medical College, Huazhong University of Science and Technology Wuhan China; ^2^ Institute of Urology Tongji Hospital, Tongji Medical College, Huazhong University of Science and Technology Wuhan China; ^3^ Department of Urology Shenzhen Hospital of Integrated Traditional Chinese and Western Medicine Shenzhen China; ^4^ Department of Urology The First Affiliated Hospital of Zhengzhou University Zhengzhou China; ^5^ Department of Integrated Traditional Chinese and Western Medicine Tongji Hospital, Tongji Medical College, Huazhong University of Science and Technology Wuhan China

**Keywords:** apoptosis, diabetes mellitus, erectile dysfunction, fibrosis, relaxin

## Abstract

**Background:**

Diabetes mellitus‐induced erectile dysfunction (DMED) responds poorly to first‐line treatments, necessitating the development of new therapeutic strategies. Relaxin‐2 (RLX‐2) plays a crucial role in protecting vascular endothelium, vasodilatation, and antifibrosis in various diseases. However, its effects and mechanisms on DMED remain unclear.

**Objectives:**

To investigate the effects and mechanisms of RLX‐2 on DMED rats in vivo and vitro.

**Methods:**

For in vivo research, 30 Sprague–Dawley rats were allocated into three groups: control, DMED, and DMED + RLX‐2. The induction of DMED in the rats was achieved through intraperitoneal administration of streptozotocin, with confirmation of ED status being conducted via the apomorphine test. Rats in the DMED + RLX‐2 group received continuous RLX‐2 treatment by osmotic pump. Following a 4‐week treatment period, assessment of erectile function was carried out using cavernous manometry, and samples of corpus cavernosum tissues were procured for subsequent analysis. For in vitro research, human cardiac microvascular endothelial cells (HCMECs) were allocated into three groups: control, high glucose (HG, 40 mM), and HG + RLX‐2. HCMECs were cultured for 6 days and treated with RLX‐2 for 48 h before collection for subsequent experiments.

**Results:**

In DMED rats, RLX‐2 treatment partially improved erectile function. We observed relatively normalized functions of endothelial and smooth muscle cells with decreased levels of apoptosis and fibrosis in the penis. In vitro experiments also demonstrated the antihyperglycemic effects of RLX‐2.

**Conclusions:**

RLX‐2 can protect endothelial and smooth muscle function, and inhibit aberrant apoptosis and fibrosis in the corpus cavernosum, thereby improving erectile function in DMED rats. This may provide a novel treatment for DMED.

## 1 INTRODUCTION

Erectile dysfunction (ED) is characterized by the inability of the penis to achieve and sustain an erection of adequate rigidity and duration for satisfactory sexual intercourse.^1^ The etiopathogenesis of ED is multifactorial, involving various metabolic abnormalities, aging, neurological injury, medications, and so on.^2^, ^3^, ^4^, ^5^ Metabolic factors such as diabetes mellitus (DM), hypertension, hyperlipidemia, obesity, and metabolic syndrome can lead to the development of ED.^2^, ^6^, ^7^, ^8^, ^9^ Erectile dysfunction has emerged as a prevalent complication of endocrine disorders, particularly diabetes mellitus. As a significant complication of male DM, the prevalence of erectile dysfunction in individuals with diabetes can reach as high as 75%. Diabetic patients often experience ED a decade earlier than the general population.^10^, ^11^ Phosphodiesterase 5 inhibitors (PDE5Is) are frequently employed as the initial therapeutic approach for ED.^12^ However, compared with non‐diabetic patients, the effectiveness of PDE5Is in diabetic patients is significantly reduced.^13^ Therefore, new therapies are urgently needed to treat DM‐induced ED (DMED) patients.

DM can affect erectile function through multiple targets. In diabetic patients, multiple factors such as corporal endothelial dysfunction, oxidative stress‐induced cavernous smooth muscle injury and dysfunction of NO‐cGMP pathway jointly promote the occurrence of DMED.^14^, ^15^, ^16^ Previously, researches on DMED treatment mainly focus on the inhibition of PDE5 activity, but regulation of a single target makes it difficult to achieve an ideal therapeutic effect. Therefore, only by regulating multiple targets that affect the etiopathogenesis of DMED can we fundamentally improve its prognosis.

Frederick Hisaw discovered relaxin in 1926 as an endocrine hormone relaxing the birth canal.^17^ The relaxin family consists of 7 polypeptides, with RLX‐2 being the predominant relaxin type present in mammalian circulation. RLX‐2 garnered attention for its multiple cardiovascular protective properties, such as vasodilatation, angiogenesis promotion, and antifibrosis.^18^, ^19^, ^20^ The transmembrane G protein‐coupled receptor RXFP1, which binds RLX‐2, is extensively expressed in cardiovascular, reproductive, renal, pulmonary, hepatic, and other organs.^21^ In vascular tissue, RXFP1 is located in smooth muscle cells and endothelial cells. RLX‐2 binds to RXFP1 to mediate multiple vascular effects mainly by activating cAMP and NO signaling.^22^ Through activating the phosphatidylinositide 3‐kinases/protein kinase B (PI3K/AKT) and endogenous nitric oxide synthase (NOS), RLX‐2 can rapidly relax blood vessels.

Though recent research on RLX‐2 mainly focuses on cardiovascular fields, several studies have shown that RXFP1 agonists have therapeutic effects in diabetic patients. They can protect vascular endothelial function, promote vascular relaxation, and resist tissue oxidative stress and fibrosis.^23^, ^24^, ^25^ Meanwhile, multiple clinical trials have validated the safety and effectiveness of relaxin in treating various diseases, including acute heart failure, renal failure, renal fibrosis, and liver injury by activating RXFP1.^26^, ^27^, ^28^, ^29^ Our prior research has shown that RLX‐2 enhanced erectile function in rats with bilateral cavernous nerve injury.^30^ However, it is not known whether RLX‐2 can treat diabetic ED.

As vascular endothelial dysfunction and pathological changes in penile corporal tissue are the core causes of DMED, whether the RLX‐2/RXFP1 pathway can serve as an effective therapeutic target for DMED deserves investigation.

## 2 MATERIALS AND METHODS

### 2.1 Animal

The study was approved by the Experimental Animal Ethics Committee of Tongji Hospital, Tongji Medical College, Huazhong University of Science and Technology, Wuhan, China (TJH‐201910005).

This study involved 32 8‐week‐old male Sprague–Dawley rats. All rats underwent 1 week acclimatization before the formal experiment. Type I diabetes mellitus model was established in 22 randomly selected rats by intraperitoneal injection of 1% streptozotocin (60 mg/kg).^31^ 10 rats, designated as the Control group, received intraperitoneal injections of the vehicle (0.1 mol/L citrate phosphate buffer; pH 4.2). Rats with fasting blood glucose levels exceeding 16.7 mmol/L after 72 h were selected as DM models.

Eight weeks later, the erectile function of rats was evaluated by subcutaneous injection of 100µg/kg apomorphine. Within 30 min after administration, rats without erections were classified as DMED rats.^32^ Subsequently, 20 DMED rats were randomly assigned to either the DMED group or the DMED+RLX‐2 group (*n *= 10). Under aseptic conditions, the DMED+RLX‐2 group received 2 mL of RLX‐2 (0.4 mg/kg/day) solution injected via osmotic pumps. In the other two groups, rats received equal amounts of sterile saline. All rats were administered a 120 TU penicillin intramuscular injection for three consecutive days as a prophylactic measure against infection. Following a 4‐week treatment period, all rats exhibited survival without any adverse events.

### 2.2 Evaluation of erectile function

Subsequent to a 3‐day washout period, the erectile function of rats was evaluated through electrical stimulation of the cavernous nerves. Bilateral isolation of the cavernous nerves was conducted, followed by electrical stimulation at 5.0 V (15 Hz; 1.2 ms, 1 min). Mean arterial pressure (MAP) and intracavernosal pressure (ICP) were measured using a pressure transducer. After evaluations, cavernous tissues were collected and embedded in paraffin wax or kept at –80°C.

### 2.3 Cell culture and evaluation of cell viability

The HCMEC cell line (WarnerBio) was derived from primary human cardiac microvascular endothelial cells by transducing SV40 and hTERT. HCMECs were cultured in six‐well plates using endothelial cell medium (ECM, ScienCell) at 37°C with 5% CO_2_.

Then HCMECs were divided into three experimental groups: Control group, HG group and HG + RLX‐2 group. The glucose concentration was 5 mM in the control group and 40 mM in the HG group and HG + RLX‐2 group. The HG + RLX‐2 group was treated with 20 nM RLX‐2 for 48 h following a6‐day high glucose exposure.

Cell viability was assessed using the Cell Counting Kit‐8 (CCK‐8) assay kit (AR1199; Boster). Following treatment, HCMECs were seeded onto 96‐well culture plates at a density of 1 × 10^4^ cells per well and incubated for an additional 24 h. Subsequently, CCK‐8 reagent was introduced to each well and allowed to incubate at 37°C for 1 h. The absorbance was quantified at a wavelength of 450 nm.

### 2.4 Western Blot

Freshly frozen tissues were used for the assessment of protein expression, followed by homogenization in RIPA lysate (R0010, Solarbio) supplemented with Protease and Phosphatase Inhibitor Cocktail (78440, Thermo Fisher Scientific). Subsequently, proteins were separated via SDS‐PAGE and transferred onto PVDF membranes. Following membrane blocking with Tris‐buffered saline‐Tween containing 5% bovine serum albumin for 1 h at room temperature, the membrane was exposed to primary antibody overnight at 4°C. Subsequent incubation with the corresponding secondary antibody for 1 h at room temperature ensued. Band visualization was conducted by the ChemiDocTM MP Imaging System. Primary antibodies used refer to Table S1.

### 2.5 Immunofluorescence (IF) and immunohistochemistry (IHC)

Paraffin sections of the penis and HCMEC slides were utilized for IF and IHC. Positive areas reflect the distributions and levels of targets. Primary antibodies used refer to Table S1.

### 2.6 Masson's trichrome staining

Paraffin sections of the penis were utilized for Masson's trichrome staining to evaluate fibrosis in penis. The area of red part reflects the components of smooth muscle. The area of blue part reflects the components of collagen.

### 2.7 Terminal deoxynucleotidyl transferase‐mediated nick end labeling (TUNEL) staining

Paraffin sections of the penis were used for TUNEL staining to evaluate apoptosis in penis. Apoptotic cell nuclei were characterized by red fluorescence.

### 2.8 Detection of nitric oxide (NO), cyclic guanosine monophosphate (cGMP) and Ca^2+^

Freshly frozen tissues of penis were applied to evaluate NO (S3090, Beyotime), cGMP (Mbbiology) and Ca2+ (S1063S, Beyotime). Concentrations were standardized based on protein concentrations.

### 2.9 Assessment of cell apoptosis

Apoptosis was evaluated by counting HCMECs using flow cytometry after pretreatment with the Annexin V‐APC/7‐AAD apoptosis kit (AP105, MultiSciences). This experiment was finished at the Experimental Medical Research Center of Tongji Hospital.

### 2.10 Detection of ROS

Reactive oxygen species (ROS) levels in human cardiac microvascular endothelial cells (HCMECs) were assessed using the H2DCFH‐DA probe (BL714A, Biosharp). The HCMEC slides were treated with a 10 µM H2DCFH‐DA probe solution and incubated for 30 min at 37°C in darkness, following the manufacturer's instructions.

### 2.11 Statistical analysis

The intensity or areas of images were calculated by ImageJ. The data was analyzed using GraphPad Prism 9.0, and statistical analyses were conducted using one‐way ANOVA and Tukey's Honest Significant Difference test with a significance level set at *p* < 0.05.

## 3 RESULTS

### 3.1 RLX‐2 did not impact metabolic parameters

**FIGURE 1:**
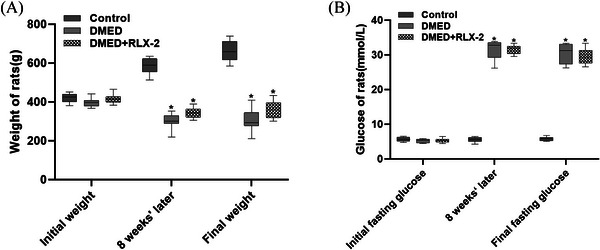
Assessment of metabolic and physiological parameters of rats. Body weight (A) and fasting blood glucose (B) in different groups (numbers of determinations for each group = 8) during the trial. *p < 0.05 of the Control group vs. the DMED group or the DMED+RLX‐2 group. DMED: diabetes mellitus‐induced erectile dysfunction; RLX‐2: relaxin‐2.

As depicted in Figure 1, the initial body weight and fasting blood glucose levels were comparable among all rats. Following an 8 weeks’ DM induction, the DM rats exhibited a significant reduction in body weight and an elevation in blood glucose levels in comparison with the control group. Moreover, this pattern persisted after a 4‐week treatment with RLX‐2. These findings suggested the successful establishment of a DM rat model.

### 3.2 RLX‐2 demonstrated an enhancement in erectile function in rats with DMED

**FIGURE 2:**
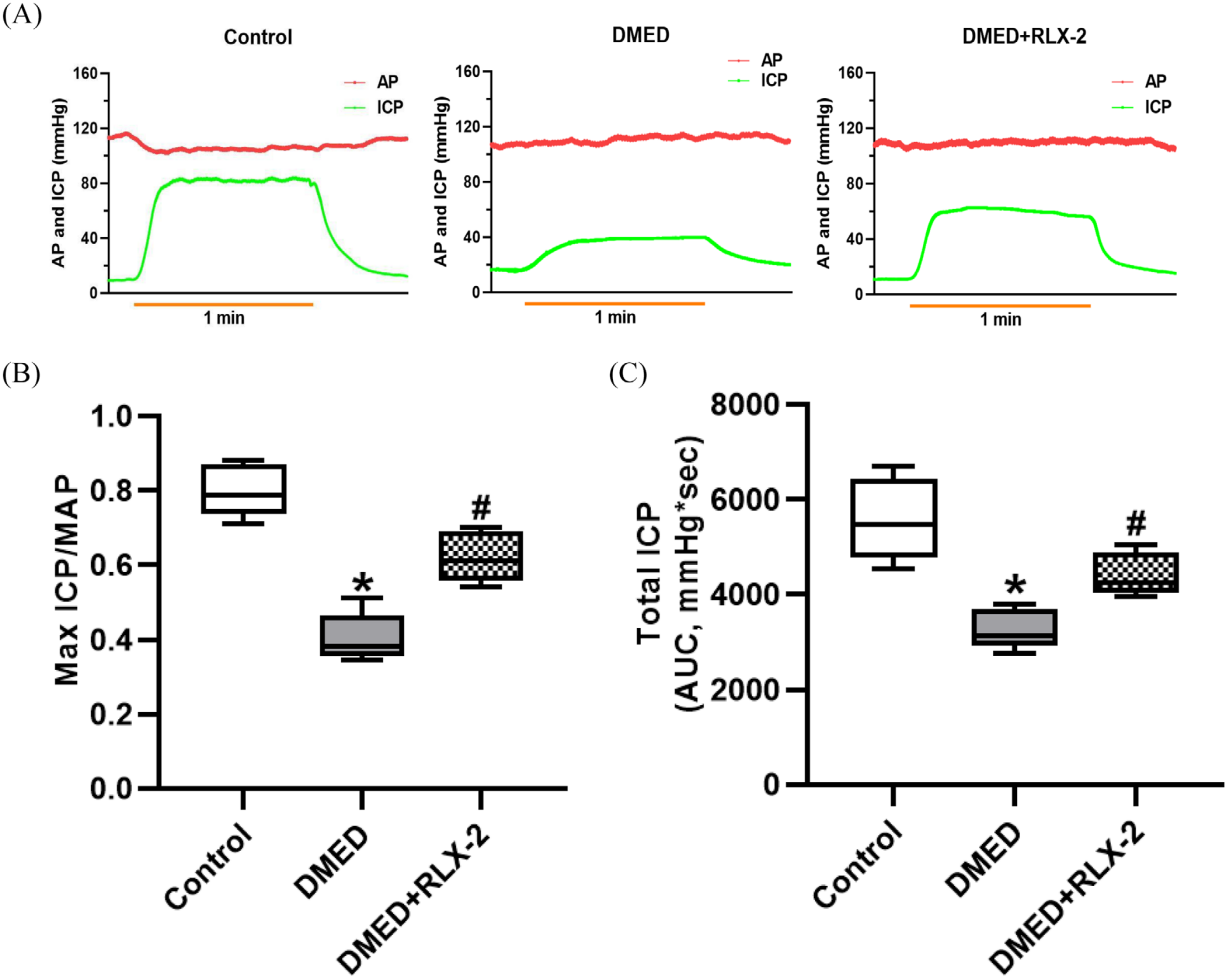
Evaluation of erectile function. (A) Representative curves of AP and ICP in the three groups (15 Hz; 1.2 ms; 5.0 V; stimulation duration 60 s;). (B) Maximum ICP/MAP and (C) AUC in the three groups (numbers of determinations for each group = 5). *p < 0.05 of the Control group vs. the DMED group; #*p* < 0.05 of the DMED+RLX‐2 group vs. the DMED group. AP: arterial pressure; ICP: intracavernosal pressure; MAP: mean arterial pressure; AUC: area under the ICP curve; DMED: diabetes mellitus‐induced erectile dysfunction; RLX‐2: relaxin‐2.

Evaluation of erectile function was conducted by stimulating the bilateral cavernous nerves of rats in all three experimental groups at 5.0 V and 15.0 Hz, with measurements of arterial pressure and ICP taken (Figure 2A). Notably, while maintaining a consistent MAP, the maximum ICP/MAP ratio and total ICP experienced a significant decrease in the DMED group but showed improvement in the DMED+RLX‐2 group (Figure 2B,C). These findings suggested that treatment with RLX‐2 may offer partial amelioration of erectile dysfunction in diabetic rats.

### 3.3 RLX‐2 protected endothelial function

**FIGURE 3:**
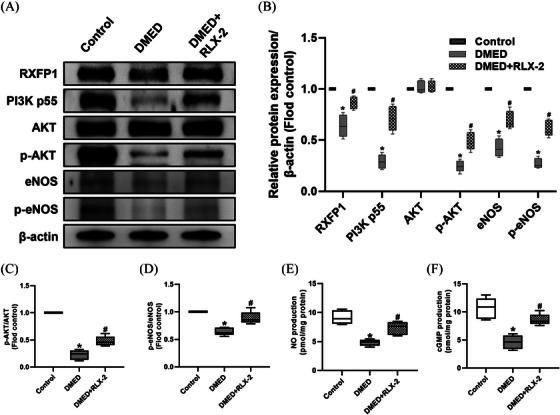
Effect of RLX‐2 on regulating RXFP1/PI3K/AKT/eNOS and NO/cGMP pathway in the corpus cavernosum of DMED rat model. Representative immunoblot (A) and semiquantification (B to D) of RXFP1, PI3K p55, AKT, phosphorylated AKT, eNOS and phosphorylated eNOS in different groups (numbers of determinations for each group = 5). Level of NO (E) and cGMP (F) in different groups (numbers of determinations for each group = 5). *p < 0.05 of the Control group vs. the DMED group; #*p* < 0.05 of the DMED+RLX‐2 group vs. the DMED group. RXFP1: relaxin family peptide receptor 1; PI3K: phosphatidylinositide 3‐kinases; AKT: protein kinase B; p‐AKT: phosphorylated AKT; eNOS: endothelial nitric oxide synthase; p‐eNOS: phospho‐eNOS; cGMP: cyclic guanosine monophosphate; RLX‐2: relaxin‐2; NO: nitric oxide; DMED: diabetes mellitus‐induced erectile dysfunction.

As illustrated in Figure 3A–D, Western blotting analysis revealed decreased expression levels of RXFP1, PI3K p55, and eNOS, as well as the reduced ratios of p‐AKT/AKT and p‐eNOS/eNOS in the DMED group, suggesting endothelial dysfunction in penis. These changes were reversed following treatment with RLX‐2, indicating that endothelial dysfunction was ameliorated. Additionally, changes in the NO/cGMP pathway, a downstream pathway of eNOS, showed similar changes (Figure 3E,F).

### 3.4 RLX‐2 suppressed dysfunction of smooth muscle

**FIGURE 4:**
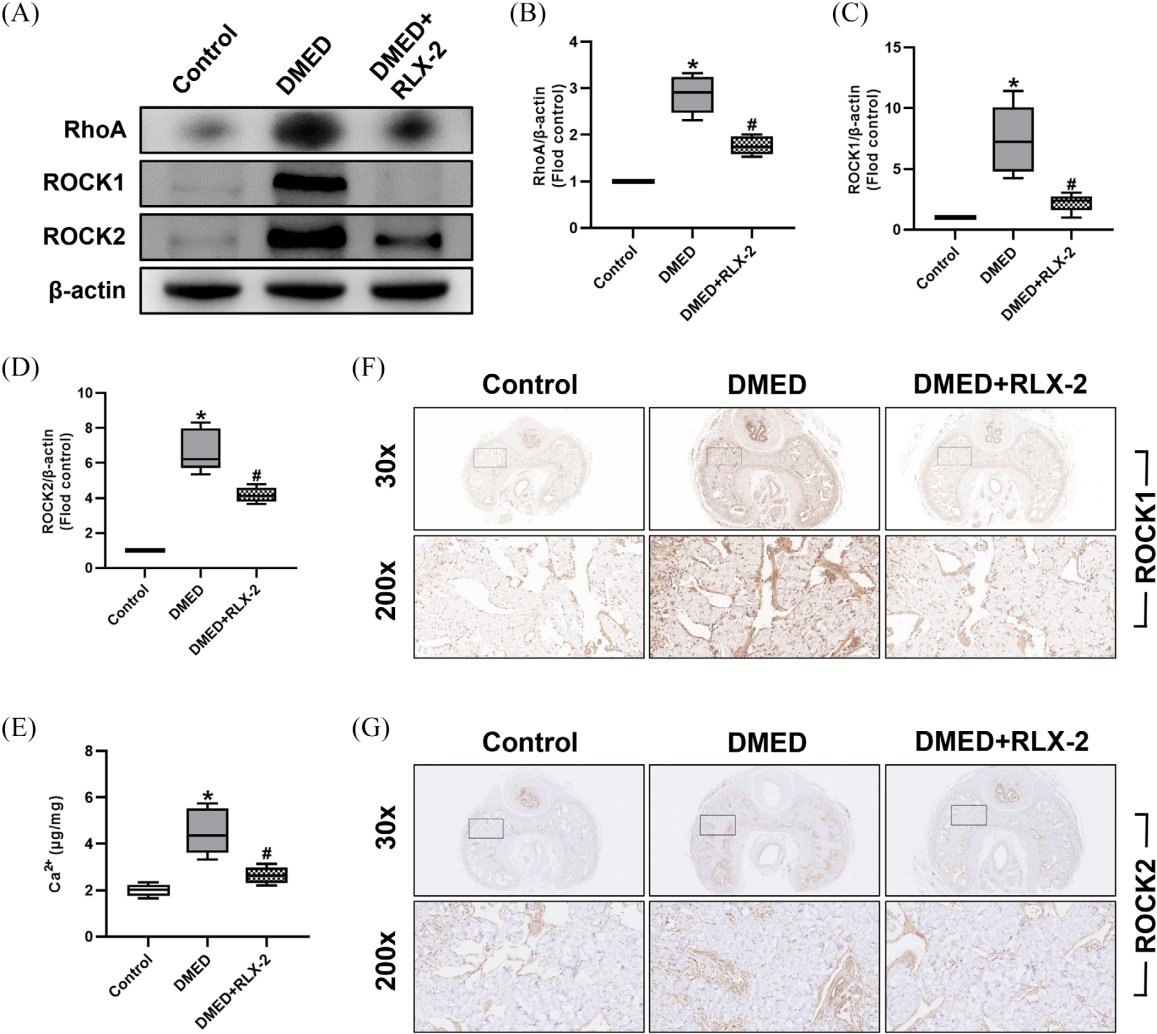
Effects of RLX‐2 on regulating the RhoA/ROCK pathway in the corpus cavernosum of DMED rat model. Representative immunoblot (A) and semiquantification (B to D) of RhoA, ROCK1 and ROCK2 in different groups (numbers of determinations for each group = 5). Level of Ca^2+^ (E) in different groups (numbers of determinations for each group = 5). Representative immunohistochemistry (×30 and ×200) of ROCK1 (F) and ROCK2 (G) in different groups. *p < 0.05 of the Control group vs. the DMED group; #p < 0.05 of the DMED+RLX‐2 group vs. the DMED group. RLX‐2: relaxin‐2; DMED: diabetes mellitus‐induced erectile dysfunction.

The findings from Western blot and immunohistochemistry analyses revealed a significant elevation in the expression levels of RhoA, ROCK1, and ROCK2 in the DMED group compared to the Control group (Figure 4A–D,F,G). Additionally, there was a notable increase in Ca^2+^ concentrations (Figure 4E). Following treatment with RLX‐2, these biomarkers exhibited a significant reduction in their levels.

### 3.5 RLX‐2 reduced fibrosis in the corpus cavernosum

**FIGURE 5:**
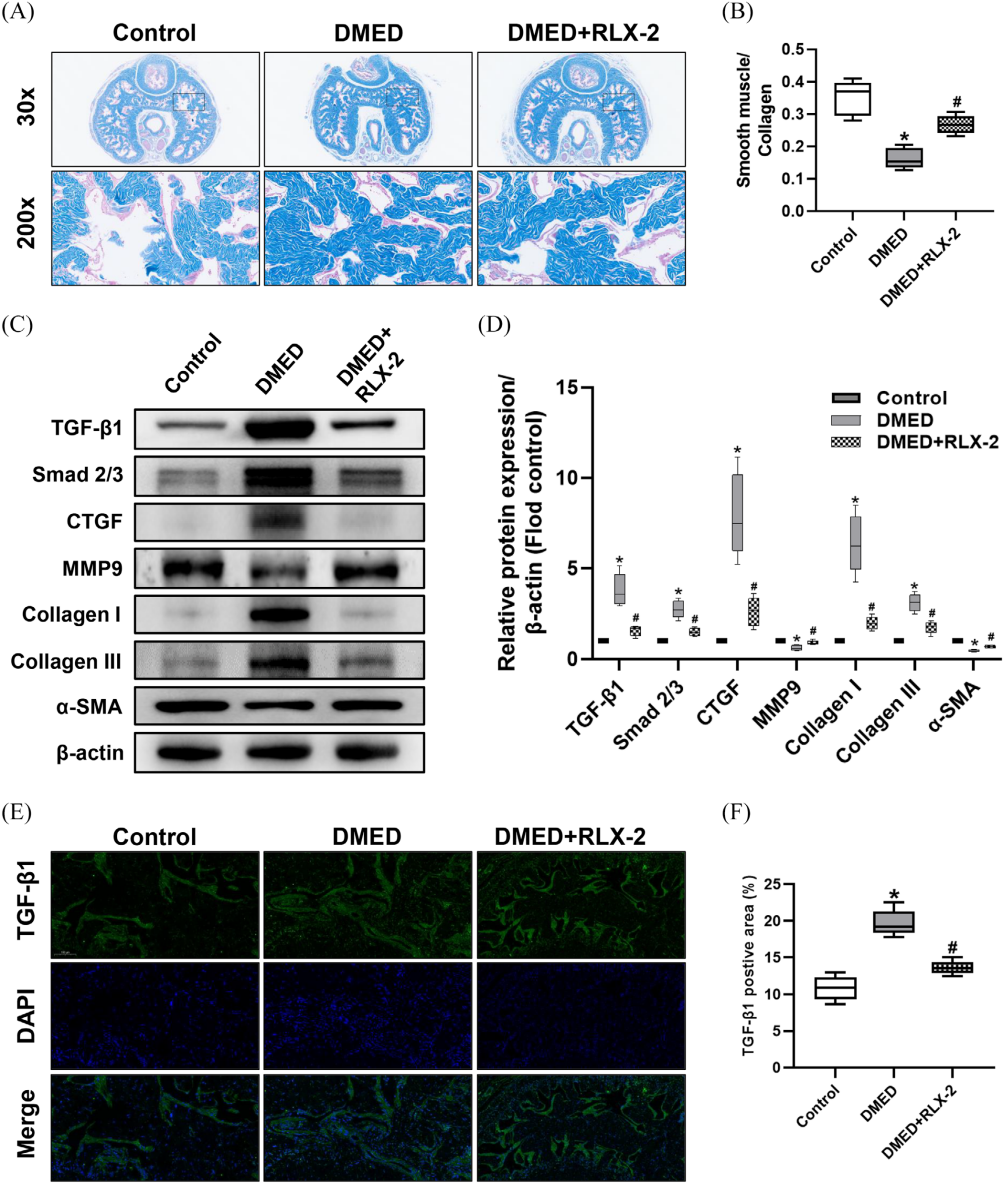
Effects of RLX‐2 on reducing fibrosis in the corpus cavernosum of DMED rat model. Representative images of Masson trichrome staining (×30 and ×200) (A) and semi‐quantification (B) in penis for each group. Representative immunoblot (C) and semi‐quantification (D) of TGFβ1, Smad 2/3, CTGF, MMP9, Collagen I, Collagen III and α‐SMA in different groups (numbers of determinations for each group = 5). Representative immunofluorescence (×200) (E) and semiquantification (F) of TGF‐β1 in different groups (numbers of determinations for each group = 5). *p < 0.05 of the Control group vs. the DMED group; #*p* < 0.05 of the DMED+RLX‐2 group vs. the DMED group. TGF‐β1: transforming growth factor β1; CTGF: connective tissue growth factor; MMP: matrix metalloprotein; α‐SMA: α‐smooth muscle actin. RLX‐2: relaxin‐2; DMED: diabetes mellitus‐induced erectile dysfunction.

As demonstrated in Figure 5A,B, Masson trichrome staining indicated a notable reduction in the smooth muscle to collagen ratio in the penis of the DMED group, which was subsequently reversed following RLX‐2 treatment, suggesting the potential of RLX‐2 in mitigating fibrosis in penis. This anti‐fibrotic effect was further supported by immunofluorescence analysis of TGF‐β1 and Western blot analysis of TGFβ1, Smad 2/3, CTGF, MMP9, Collagen I, Collagen III, and α‐SMA (Figure 5C–F).

### 3.6 RLX‐2 inhibited apoptosis in vivo

**FIGURE 6:**
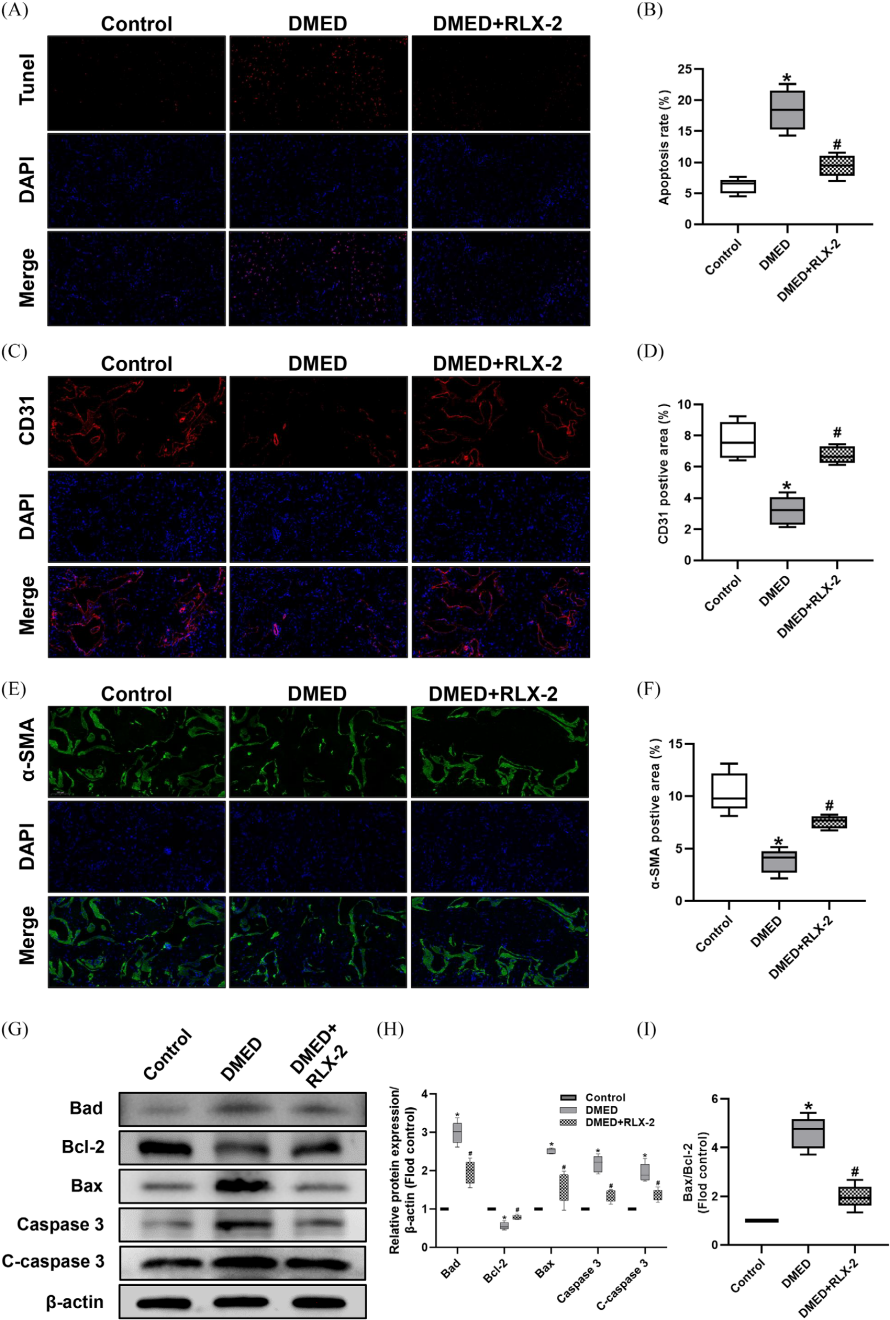
Effects of RLX‐2 on apoptosis in the corpus cavernosum of DMED rat model. Representative images of TUNEL staining (×30 and ×200) (A) and semi‐quantification (B) in penis for each group (numbers of determinations for each group = 5). Representative immunofluorescence (×200) (C and E) and semi‐quantification (D and F) of CD31 and α‐SMA in different groups (numbers of determinations for each group = 5). Representative immunoblot (G) and semi‐quantification (H and I) of Bad, Bcl‐2, Bax, Caspase 3 and C‐caspase 3 in different groups (numbers of determinations for each group = 5). *p < 0.05 of the Control group vs. the DMED group; #*p* < 0.05 of the DMED+RLX‐2 group vs. the DMED group. TUNEL = Terminal deoxynucleotidyl transferase‐mediated nick end labeling staining; C‐caspase‐3: Cleaved caspase‐3. RLX‐2: relaxin‐2; DMED: diabetes mellitus‐induced erectile dysfunction.

According to TUNEL staining analysis, the DMED group exhibited the highest level of apoptosis which subsequently decreased following RLX‐2 treatment (Figure 6A,B). Endothelial cells and smooth muscle cells are recognized as the principal effector cells within the corpus cavernosum. Likewise, the expressions of their respective markers, CD31 and α‐SMA, experienced a notable decrease in the DMED group but were restored post RLX‐2 treatment (Figure 6C–F). Additionally, the expressions of Bad, Caspase‐3, cleaved Caspase‐3, and the Bax/Bcl‐2 ratio also indicated that RLX‐2 may mitigate apoptosis in vivo (Figure 6G–I).

### 3.7 RLX‐2 reduced high glucose‐induced apoptosis in HCMECs

**FIGURE 7:**
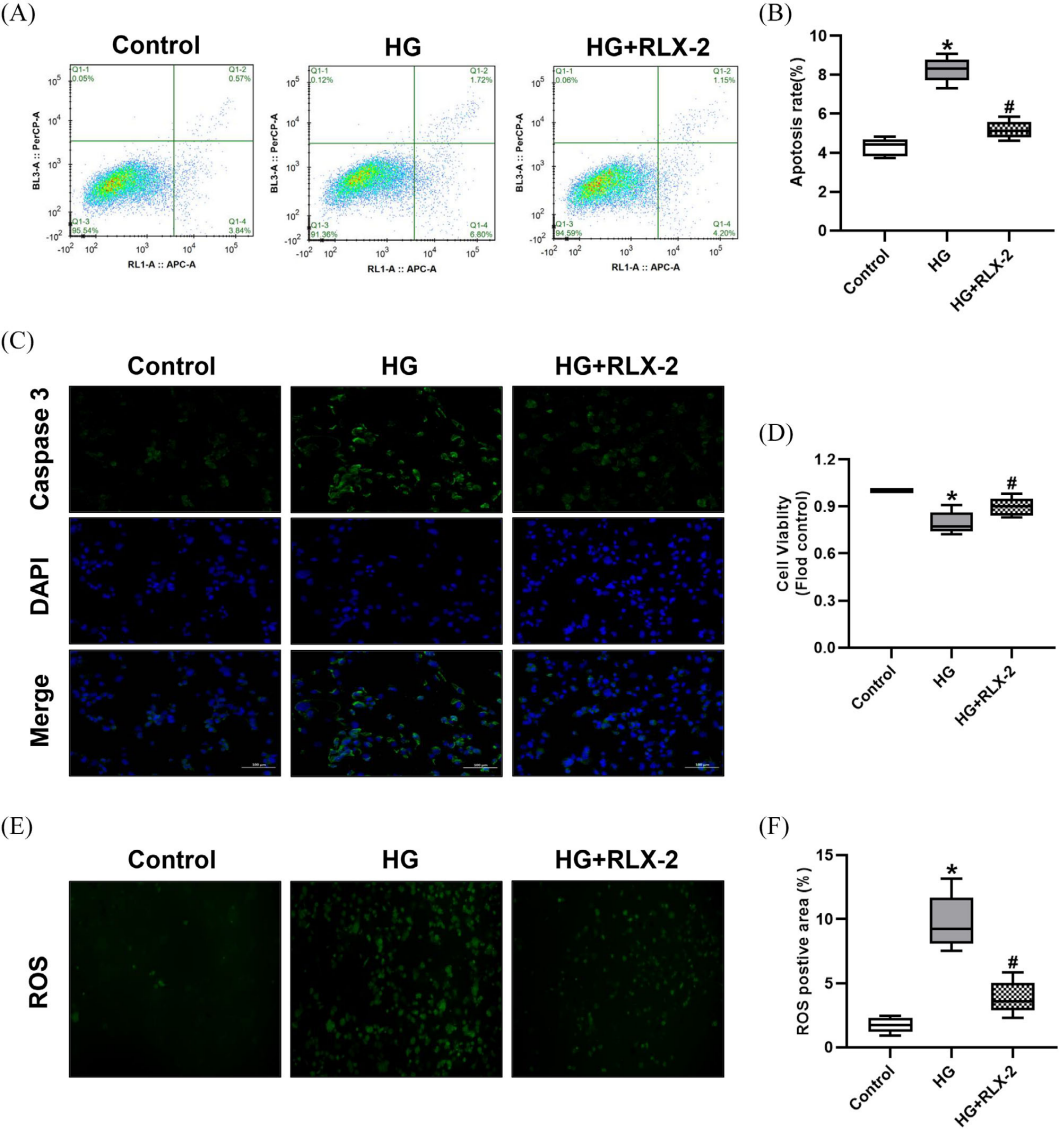
Effects of RLX‐2 on reducing apoptosis induced by high glucose in HCMECs. Representative data of flow cytometry (A) and comparisons of apoptosis rate (B) in HCMECs among the groups (numbers of determinations for each group = 5, from 5 different cultures). Representative immunofluorescence (×200) (C) of caspase 3 in HCMECs among the groups. The results of cell viability assay (D) in different groups (numbers of determinations for each group = 7, from 7 different cultures). Representative fluorescence (×100) (E) and semi‐quantification (F) of ROS in HCMECs among the groups (numbers of determinations for each group = 5, from 3 different cultures). *p < 0.05 of the Control group vs. the HG group; #p < 0.05 of the HG+RLX‐2 group vs. the HG group. HCMEC: Human Cardiac Microvascular Endothelial Cells; ROS: reactive oxygen species; HG: high glucose; RLX‐2: relaxin‐2.

As illustrated in Figure 7A,B, apoptotic percentage was significantly elevated in the HG group, but decreased following treatment with RLX‐2. This observation was further supported by immunofluorescence analysis of Caspase 3 (Figure 7C). Additionally, RLX‐2 treatment was associated with enhanced cell viability (Figure 7D) and reduced levels of ROS (Figure 7E,F).

## 4 DISCUSSION

Diabetes mellitus is a metabolic disorder characterized by vascular dysfunction, which can also affect the penis as part of the circulatory system, resulting in DMED. Due to the limited therapeutic effect of the first‐line therapeutic drug PDE5I, DMED has been a significant concern for patients and doctors worldwide. Given the strong regulatory ability of RLX‐2 in pathological environments,^33^, ^34^, ^35^ we used RLX‐2 to improve DMED and researched its potential mechanism. The findings indicate that RLX‐2 may enhance erectile function in diabetic rats by preserving endothelial and smooth muscle function, as well as inhibiting cavernous fibrosis and apoptosis. In addition, our study also revealed that RLX‐2 could inhibit HCMEC apoptosis, reduce ROS levels, and improve cell viability under HG conditions in vitro.

The PI3K/AKT/eNOS pathway plays a crucial role in regulating endothelial function and physiological erection.^36^ Studies showed that the maintenance of penile erection depends on AKT phosphorylation.^36^, ^37^ Phosphorylated AKT can activate eNOS, leading to the synthesis and release of NO. The reduction in phosphorylation levels of AKT and eNOS in the corpus cavernosum of rats may contribute to ED. Pior research has demonstrated that RLX‐2/RXFP1 has the ability to stimulate eNOS activation via extracellular signal‐regulated kinases, Gβγ subunits, and 3‐phosphate inositol pathways, thereby increasing NO levels.^38^ In the present study, it was observed that the expression of RXFP1, PI3K subunit P55, phosphorylation levels of AKT and eNOS were reduced in the corpus cavernosum of DMED rats. After RLX‐2 treatment, improvements were observed, indicating that RLX‐2 may enhance PI3K/AKT activation via RXFP1, leading to increased eNOS phosphorylation, NO synthesis and release, and ultimately an improvement in erectile function.

The RhoA/ROCK pathway plays a crucial role in regulating cavernous smooth muscle contraction. ROCK can inactivate MLCP by phosphorylating myosin phosphatase‐targeting subunit 1 (MYPT‐1), and then cause contraction of corpus cavernosum smooth muscle, finally leading to ED.^39^ At the same time, RhoA can also reduce NO production by inhibiting eNOS activity, thereby downregulating NO/cGMP pathway activity and inhibiting erection. This research demonstrates that RhoA/ROCK pathway was overexpressed in diabetic rats and could be normalized through supplementation with RLX‐2.

Fibrosis within the corpus cavernosum is an influential histopathological change in DMED. It is primarily identified by the excessive accumulation of extracellular matrix and collagen fibers, accompanied by a reduction in smooth muscle. Research has indicated that the activation of the TGF‐β1/Smad/CTGF signaling cascade is a key contributor to the development of corpus cavernosum fibrosis in the context of DMED.^40^ Studies on diseases such as myocardial fibrosis have shown that RLX‐2 can inhibit endothelial cell transformation into mesenchymal cells by activating the RXFP1/eNOS/NO pathway, thereby downregulating TGF‐β1/Smad/CTGF signaling and exerting antifibrotic effects.^41^ In this study, we further validated the role of TGF‐β1/Smad/CTGF pathway in corpus cavernosum fibrosis induced by DMED. The experimental results also showed that RLX‐2 can reduce collagen deposition and degrade fibrotic tissue by downregulating TGF‐β1/Smad/CTGF pathway and increasing MMP9 levels. These findings indicated that RLX‐2 may possess anti‐fibrotic effects in corpus cavernosum of DMED rats.

In DM rats, excessive apoptosis in penis can lead to tissue dysfunction and further cause ED. Hyperglycemia can promote the accumulation of advanced glycation end products and ROS, inhibit the activity of superoxide dismutase, reduce the concentration of NO, and activate NADPH oxidase‐mediated oxidative stress reaction, leading to excessive apoptosis in the corpus cavernosum.^42^ The Bcl‐2 protein family plays a crucial role in regulating apoptosis within the signal transduction pathway, encompassing both pro‐apoptotic and anti‐apoptotic proteins.^43^ Caspase3 is a common downstream effector in various apoptotic pathways, which is influenced by the Bcl‐2 protein family. Upon activation, caspase3 cleaves DNA‐dependent protein kinase and poly ADP‐ribose polymerase, subsequently impacting DNA replication, transcription, and repair processes, ultimately culminating in apoptosis. In the present investigation, it was observed that the expression levels of pro‐apoptotic proteins Bad and Bax were increased, whereas the expression level of the anti‐apoptotic protein Bcl‐2 was decreased in the penile tissue of diabetic rats. This phenomenon is likely attributable to oxidative stress induced by hyperglycemia. After RLX‐2 treatment, these changes were improved, and excessive apoptosis in penis of DM rats was inhibited. In addition, we noticed that the apoptosis level and oxidative stress level were significantly increased in HCMEC cultured in HG environment, and RLX‐2 treatment alleviated these changes. These results further illustrated the anti‐apoptotic effect of RLX‐2.

Our research provided evidence that RLX‐2 can improve erectile function in DM rats, but there are some limitations. Given the limited impact of the diabetic model on rat androgen levels and inflammatory status, we did not measure the corresponding parameters. We used some of the key molecules rather than functional studies with cavernous tissue to characterize endothelial and smooth muscle function. We showed the potential mechanism of short‐term use of RLX‐2 for DMED, but the benefits and safety of long‐term RLX‐2 treatment is still unclear. At the same time, we only used type 1 DM models, and did not use type 2 DM models. Our results may not be extended to ED caused by type 2 DM. These limitations require further exploration in our future research.

## 5 CONCLUSIONS

Our study demonstrated that RLX‐2 has the potential to enhance erectile function in DM rats, and the potential therapeutic effect was related to preservation of endothelium and smooth muscle function, as well as suppression of cavernous fibrosis and apoptosis.

## AUTHOR CONTRIBUTIONS

Yang Luan conceived and designed the present study. Bocheng Tu contributed to formal analysis, data curation, investigation, methodology and manuscript writing. Bo Wen, Kang Liu, Peng Hu, Taotao Sun and Beining Li performed the investigation, formal analysis, and result visualization. Manan Sulaiman, Shujun Jiang, Tao Wang, and Jihong Liu carried out project administration and manuscript revising. All authors have read and approved the final manuscript.

## CONFLICT OF INTEREST STATEMENT

The authors declare no conflicts of interest.

## Supporting information



Supporting Information

## Data Availability

The data that support the findings of this study are available from the corresponding author upon reasonable request.
