# Supplementary material for: Relaxin‐2 improves type I diabetes mellitus‐induced erectile dysfunction in rats by protecting cavernous endothelial and smooth muscle function, and inhibiting penile fibrosis and apoptosis
Source: Andrology. 2024 Dec 9;13(7):1935–46. doi: 10.1111/andr.13822 (PMC12476221; doi:10.1111/andr.13822)
Supplement: Supplementary file 1 — Supporting Information [file ANDR-13-1935-s001.docx]

**Supplementary Information**

**Supplementary Table 1** The details of the antibodies used in the study.

|  | Name | Abbreviation | concentration | source |
| --- | --- | --- | --- | --- |
| WB | anti-RXFP1 | RXFP1 | 1:1000 | 44621; Signalway Antibody, MD, USA |
|  | anti-PI3K p55 | PI3K p55 | 1:1000 | 27035-1-AP; Proteintech, Wuhan, China |
|  | anti-AKT | AKT | 1:1000 | 9272; Cell Signaling Technology, Dan vers, MA, USA |
|  | anti-phosphor-AKT | phosphor-AKT | 1:1000 | 1:1,000; 4060; Cell Signaling Technology, |
|  | anti-eNOS | eNOS | 1:1000 | ab300071; Abcam, Waltham, MA, USA |
|  | anti- phospho-eNOS | phospho-eNOS | 1:1000 | 1:1,000; 9571; Cell Signaling Technology |
|  | anti-RhoA | RhoA | 1:1000 | 10749-1-AP; Proteintech |
|  | anti-ROCK1 | ROCK1 | 1:1000 | 21850-1-AP; Proteintech |
|  | anti-ROCK2 | ROCK2 | 1:1000 | 21645-1-AP; Proteintech |
|  | anti-TGF-β1 | TGF-β1 | 1:1000 | 21898-1-AP; Proteintech |
|  | anti-Smad2/3 | Smad2/3 | 1:1000 | 8685; Cell Signaling Technology |
|  | anti-CTGF | CTGF | 1:1000 | 25474-1-AP; Proteintech |
|  | anti-MMP9 | MMP9 | 1:1000 | 10375-2-AP; Proteintech |
|  | anti-Collagen Ⅰ | Collagen Ⅰ | 1:1000 | 14695-1-AP; Proteintech |
|  | anti-Collagen Ⅲ | Collagen Ⅲ | 1:1000 | 22734-1-AP; Proteintech |
|  | anti-α-SMA | α-SMA | 1:1000 | 1:1,000; GB111364; Servicebio, Wuhan, China |
|  | anti-Bad | Bad | 1:1000 | A1593; ABclonal, Wuhan, China |
|  | anti-Bcl2 | Bcl2 | 1:1000 | 12789-1-AP; Proteintech |
|  | Anti-Bax | Bax | 1:1000 | A0207; ABclonal |
|  | anti-Caspase-3 | Caspase-3 | 1:1000 | AF6311; Affinity Biosciences, Houston, TX, USA |
|  | anti-Cleaved Caspase-3 | Cleaved Caspase-3 | 1:1000 | A19654; ABclonal |
|  | anti-β-actin | β-actin | 1:1000 | GB11001; Servicebio |
| IHC | anti-ROCK1 | ROCK1 | 1:200 | 21850-1-AP; Proteintech |
|  | anti-ROCK2 | ROCK2 | 1:200 | 21645-1-AP; Proteintech |
| IF | anti-TGF-β1 | TGF-β1 | 1:500 | 21898-1-AP; Proteintech |
|  | anti-CD31 | CD31 | 1:100 | GB113151; Servicebio |
|  | anti-α-SMA | α-SMA | 1:500 | GB111364; Servicebio |
|  | anti-Caspase 3 | Caspase 3 | 1:200 | AF6311; Affinity Biosciences |

WB = western blotting; IHC = immunohistochemistry; IF = immunofluorescence

**Supplementary Figure 1** The effects of 2-day RLX-2 treatment on normoglycemia in HCMECs.

**
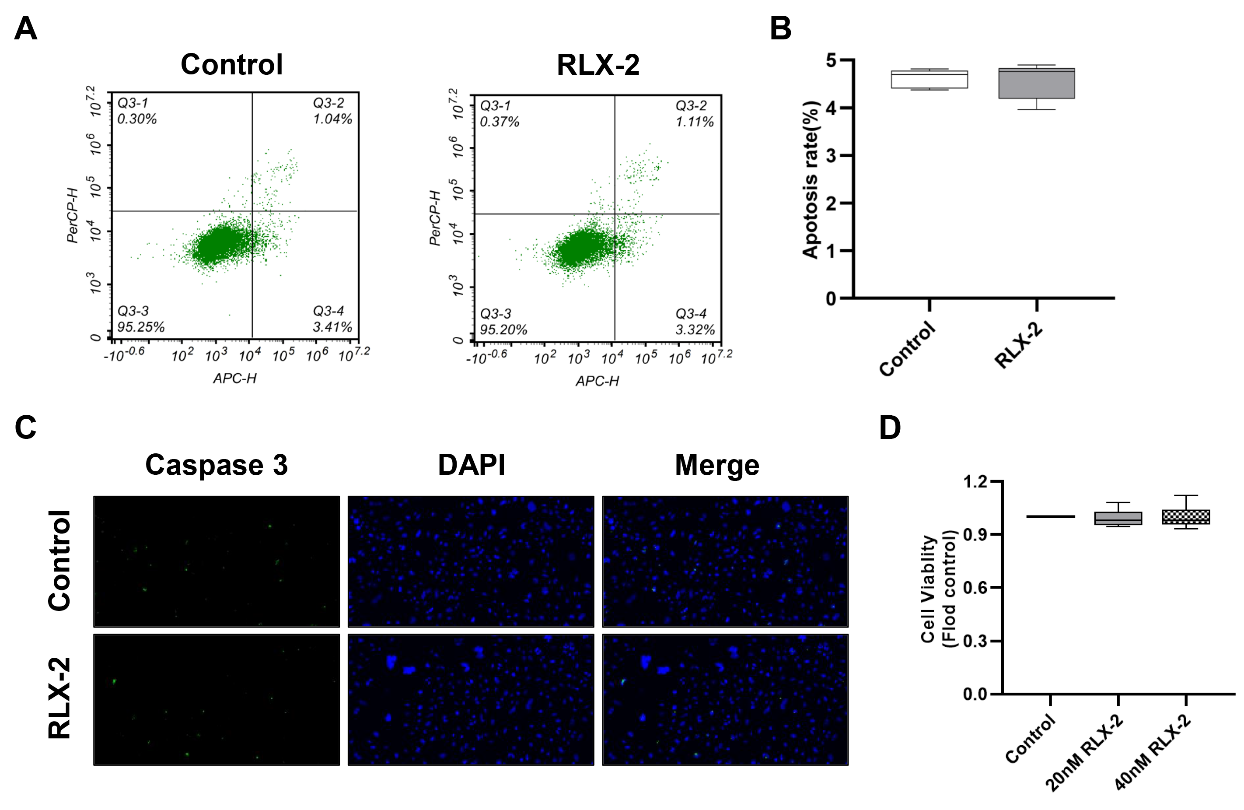
**

Representative data of flow cytometry (A) and comparisons of apoptosis rate (B) in HCMECs among the groups (numbers of determinations=5). Representative immunofluorescence (×200) (C) of caspase 3 in HCMECs among the groups. The results of cell viability assay (D) in different groups (numbers of determinations=7). The RLX-2 concentration is 20nM in A-C. HCMEC: Human Cardiac Microvascular Endothelial Cells; RLX-2: relaxin-2.

One-way ANOVA showed no significant difference between the different groups (p>0.05), indicating the safety of RLX-2.
